# Three-Dimensional Analysis of the Maxillary Sinuses in Ancient Crania Dated to the V–VI Centuries BCE from Opi (Italy): Volumetric Measurements in Ancient Skulls from the Necropolis of Opi, Abruzzi, Italy

**DOI:** 10.3390/diagnostics14151683

**Published:** 2024-08-03

**Authors:** Felice Festa, Ruggero D’Anastasio, Stefano Benazzi, Monica Macrì

**Affiliations:** 1 Department of Innovative Technologies in Medicine & Dentistry, University “G. D’Annunzio” of Chieti-Pescara, 66100 Chieti, Italy; ffesta@unich.it; 2 Department of Medicine and Aging Sciences, University ‘G. D’Annunzio’ of Chieti-Pescara, 66100 Chieti, Italy; 3 Department of Cultural Heritage, University of Bologna, 40126 Bologna, Italy

**Keywords:** upper airway, maxillary sinus, maxillary sinus volume, maxillary sinus size, maxillary pneumatisation, facial height, bizygomatic width, palatal length

## Abstract

**Simple Summary:**

Twenty ancient crania dated to the V–VI centuries from the necropolis of Opi, Abruzzi, Italy, were analysed. A 3D analysis of the maxillary sinuses of these ancient crania was performed. The aims of this paper were two: to compare the linear and volumetric measurements of the left and right maxillary sinuses and to determine the facial measurements in the same sample (the upper facial height, the bizygomatic width, and the palatal length).

**Abstract:**

Cone beam computed tomography (CBCT) can provide precise information about complex anatomical structures as it is characterised by rapid volumetric image acquisition with high resolution. The aim of this study was to provide measurements for 20 ancient skulls of the Samnite people found in the necropolis of Opi, a small and isolated mountain village in Abruzzo, a region in central Italy. All the images (left and right) of the 20 ancient skulls from Opi were acquired. All the data are the property of the Department of Innovative Technologies in Medicine and Dentistry of G. D’Annunzio University, Chieti, and different parameters (area and volume) were measured and evaluated. The mean and standard deviation of the facial measurements were also calculated. All the data were subjected to statistical analysis. CBCT scan data of 20 fossil skulls did not show significant values regarding the MS area and the volume between the right and left sides. In the ancient skulls, no difference was found between the right and left sides.

## 1. Introduction

The maxillary sinuses (MSs) are the biggest paranasal sinuses, also named the “antrum of Highmore” after being described by an English anatomist, Nathaniel Highmore, in 1651 [1]. Leonardo da Vinci described the maxillary sinuses for the first time in 1489, drawing them as symmetrical, pyramid-shaped cavities in the left and right maxillary bones [2].

A similar link between MS size and head circumference has been discovered in the fetal development of the human species [3].

Knowledge of the anatomy and growth of the orofacial complex is indispensable for formulating diagnoses and treatment plans in orthodontics [4].

Growth of the orofacial complex can be defined as a set of growth processes with different timing and modalities and modulated by functional stimuli generated by soft tissues according to Moss’s Functional Matrix Theory, which sees functional adaptation as the most influential mechanism in the architecture of the facial skeleton [5]. Growth is, therefore, a multifactorial process regulated by genetic, neurological, and hormonal factors placed under the influence of functions (breathing, swallowing, chewing, phonetics, etc.) and proceeds in three fundamental moments: ossification, direct and indirect organisation (defined as the ability of the various anatomical structures to grow in a harmonious relationship with the others), and functional adaptation [6].

Bone remodelling promotes MS expansion, which is closely related to maxillary development [7]. The MSs grow at the expense of maxillary processes due to the physiological process of maxillary pneumatisation [8].

The MSs tend to appear at the end of the second embryonic month and are completed by 18 to 20 years [9,10].

The shape and size of the MS varies amongst individuals, between genders, and in various populations [11]. The MSs stabilise after the second decade of life in women and in the third decade in men [12].

Most studies have shown no change in sinus volume with dentition status and a decrease in volume with advancing age [13].

Considering the complex structure of the MS, diagnostic methods like magnetic resonance imaging (MRI) and computed tomography (CT) are used as the gold standard to evaluate the actual anatomy of the sinuses [14]. However, their use is limited by high cost, radiation dose, and restricted accessibility. With the introduction of CBCT, these drawbacks have been overcome [15].

The volume of the MS is typically 15 cc; however, this value may be correlated with age, gender, and ethnicity [16]. Furthermore, MS volume could fluctuate due to tooth loss or absence [17]. Identifying precise linear and volumetric measurements for the MSs is clinically essential as part of the diagnostic phase before various treatment procedures. Cone beam computed tomography (CBCT) has been widely used to assess MS anatomy, particularly before dental implant rehabilitation [18].

The MS is an important anatomical structure with significant demographic variability. The first aim of this paper was to compare the linear and volumetric measurements of the left and right MSs; the second aim was to determine the facial measurements in the same sample: the upper facial height, the bizygomatic width, and the palatal length.

Since the literature on this subject is minimal, the current study was performed to determine the linear and volumetric measurements of the maxillary sinuses with CBCT for a sample of a population from the same geographic area to obtain a reliable result. This paper examines the maxillary sinuses due to the high variability among the various hominin taxa.

T studies could be conducted on the topic, comparing the ancient skulls with those of the current population of the town of Opi to know the evolution of the paranasal sinuses in the same local population.

## 2. Materials and Methods

The present paper retrospectively determined the volume of the MSs in 20 ancient fossil crania from the same necropolis in Opi.

Opi is an Italian town of 379 inhabitants in the province of L’Aquila in Abruzzo. The town is included in the protected area of the Lazio and Molise National Park in Abruzzo.

The archaeological excavations conducted in 1994 and 1996 at the entrance of the Fondillo Valley have brought to light a necropolis. The necropolis was composed of more than 153 inhumation tombs, most of them arranged in a circle and therefore reproducing the family clans to which they belonged. The entire site was dated around the V-VI centuries BCE and includes burials of individuals of different sex and age who died at different times and being bearers of distinct social roles and economic status.

This research was conducted on well-preserved skull fossils from the Samnite population found in the archaeological site of Opi (V–VI centuries BCE) [19].

The Samnites were an ancient Italic people settled in the south–central area of the peninsula. The historical region of Samnio, corresponding to part of the current regions of Abruzzo, Molise, Campania, and some marginal areas of Lazio, Puglia, and Basilicata, takes its name from this population.

The Samnites mainly practiced pastoralism and agriculture. The territory in which they were settled was mountainous and poor in resources. They were also good warriors who often clashed with the Romans for the supremacy of the territory.

Among the many wars supported by the Samnites, those against the Romans marked their disappearance and their end as a tribal entity.

**Figure 1:**
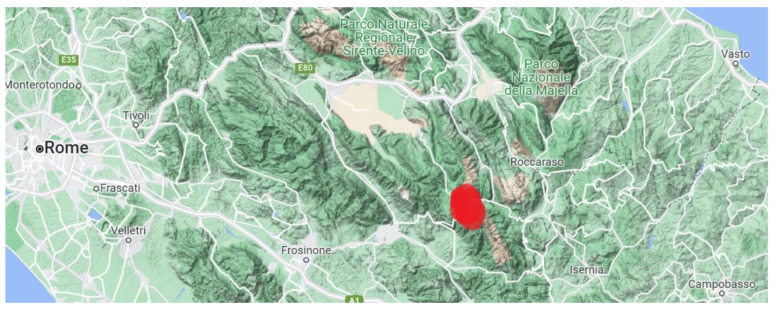
Geolocalisation (in red) of Opi (Aq) in Italy.

The analysis of skeletal and dental epigenetic traits under strong genetic control and benign neoplasias with a hereditary component made it possible to deduce that the men buried in the same funerary circle shared a kinship relationship, as reported by Viciano et al. [20]. The biological homogeneity of the Samnite population was related to the presence of strong endogamic clans or family groups due to the area being isolated by the mountains where they lived for a long time [21] (Figure 1).

The ancient Opi skulls are preserved in the National Archaeological Museum of Abruzzo, located in Chieti, Abruzzo, Italy. It was possible here, under authorisation from the University of Chieti, to select the skulls suitable for research and falling within the inclusion criteria. The recruitment period ran from July to September 2023. In this period, it was possible to select skulls suitable for the research, carry out the CBCT scans in the Department of Innovative Technologies in Medicine and Dentistry, G. D’Annunzio University of Chieti–Pescara, and proceed with the measurements.

All the data included in this work are the property of G. D’Annunzio University of Chieti–Pescara. The studies adhered to the European Union Good Practice Rules according to the Helsinki Declaration. An independent ethics committee approved the protocol (number 23) in the Hospital of Chieti.

### 2.1. Participants

The sample comprised 20 adult skulls of the Samnite population found in the necropolis of Opi (V–VI centuries BCE) to ensure a homogeneous sample in terms of ethnicity.

The following inclusion criteria were applied:

1. Skulls from the necropolis of Opi;
2. Good state of conservation of the skulls;
3. Presence of a complete denture.

Exclusion criteria were midfacial injuries, skeletal system anomalies, and missing teeth. Additionally, scans displaying MSs with unilateral abnormalities were excluded from the investigation.

### 2.2. CBCT Scans

A cone beam computed tomography (CBCT) scan was taken with the same equipment under the same conditions.

CBCT can visualise and provide precise information about teeth and surrounding complex anatomical structures as it is characterised by rapid volumetric image acquisition with high resolution and low radiation dose levels [22].

All CBCT exams were performed with a low-dose protocol (FOV of 240 × 190, acquisition time of 15 s, 80 kV, 5 mA, 35 μSv) using the Planmeca Promax^®^ 3D MID unit (Planmeca Oy, Helsinki, Finland) [23].

All the skulls examined with the CBCT technique were oriented in a natural head position (NHP), a reproducible and physiological head posture in space. The skulls were stabilised with ear rods in the external auditory meatus to obtain the NHP [24].

The CBCT data acquired were managed with Dolphin Imaging 3D software 12.0 (Dolphin Imaging and Management Solutions, Chatsworth, CA, USA), which permitted an additional head orientation on the front, right, and left views. The MS measurements were correctly performed using the widgets present in the software.

CBCT is a three-dimensional exam that allows for a more accurate morphometric analysis compared to two-dimensional exams such as latero-lateral teleradiography of the head. The accuracy of the CBCT scans allowed us to detect radiographically the boundaries of the MSs.

### 2.3. Facial Measurements

The skull analysis started with detecting three linear measurements of the face, and the values are reported in Table 1.

The facial measurements included the upper facial height, the bizygomatic width, and the palatal length.

**Figure 2:**
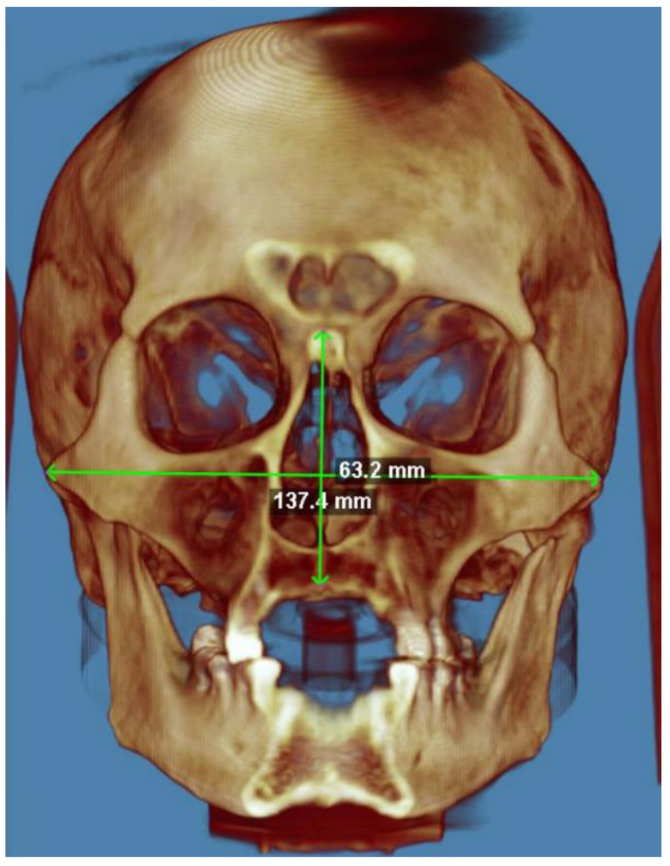
Upper facial height and bizygomatic width.

Upper facial height (Figure 2) is a linear measurement that indicates the distance from the nasion to the prosthion and is measured with a straight compass.

The nasion is the meeting point of the median sagittal plane with the nasofrontal suture. The prosthion is the most protruding point of the superior alveolar process (a few mm above the superior infradentale). The prosthion must be precisely identified as it is often confused with the upper infradentale.

Bizygomatic width (Figure 2) is a linear measurement that indicates the distance between the two “zighion” points and is calculated using a curved compass. The zighion is the most prominent point on the external surface of the zygomatic arch.

**Figure 3:**
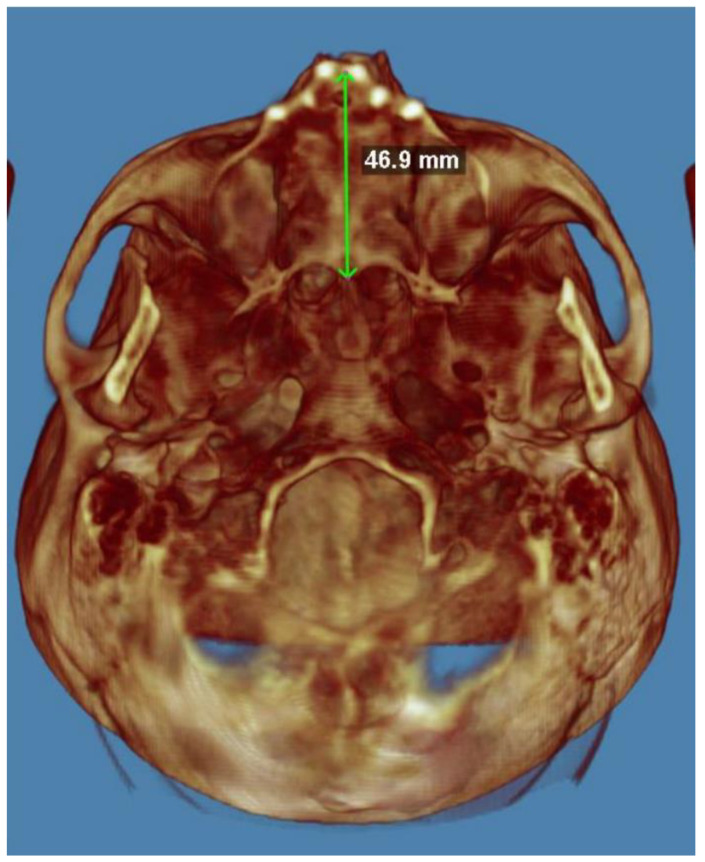
Palatal length.

Palatal length (Figure 3) is a linear measurement that indicates the projective distance between the “oral” and the “stafilion” and is measured with a straight-branched compass. The oral is the anterior point of the hole of the anterior palatine duct. The stafilion is located on the apex of the palatine spine.

### 2.4. Maxillary Sinus Measurement and Volume Determination

**Figure 4:**
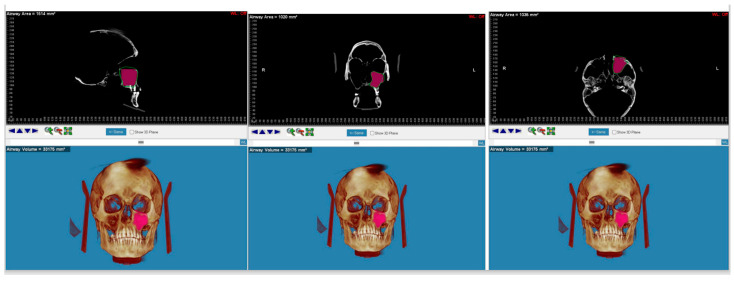
CBCT detection of maximum linear measurement along the sagittal axis, transversal axis, and vertical axis.

In the CBCT images, it was possible to visualise and measure the volume of the maxillary sinuses and their area in all three planes of space (Figure 4).

### 2.5. Error Method

This work’s reliability was improved by randomly analysing and selecting all the CBCT images. At most, 10 scans/die were checked to reduce the risk of inaccurate analysis due to fatigue. Two different observers checked all the data twice and performed all linear and volumetric measurements; in case of disagreement, a third operator was consulted to reduce intraoperator and interoperator errors. Cohen’s* Kappa determined the reliability and showed a strong significant agreement (k = 0.84) between the measurements performed by the two observers.

### 2.6. Statistical Analyses

The statistical analysis regarding the facial measurements was performed using the Kolmogorov–Smirnov test of normality.

The data analysis started with a t-test, which did not yield reliable results.

The Shapiro–Wilk and Jarque–Bera tests were used to verify the presence of a symmetric and normal distribution of the data. Both the Jarque–Bera test and the Shapiro–Wilk test returned negative results. This implies that the distribution of observations (of the samples) significantly deviates from the Gaussian distribution.

The non-parametric statistical Wilcoxon test was used to compare two paired samples. This test is particularly useful when the data do not meet the normality assumptions required for parametric tests.

The Wilcoxon test was repeated for four sets of measurements: volumes of the right and left maxillary sinuses and their respective measurements along the three axes of space at their maximum extent.

The null hypothesis (H0) of the Wilcoxon test states that there is no statistical difference between the means of the two samples. In contrast, the alternative hypothesis (H1) suggests a significant statistical difference. The significance level (α) was set at 0.05 to support the alternative hypothesis (H1); if the *p*-value is lower than the chosen significance level, the null hypothesis is rejected. If the *p*-value is higher than the significance level, there is insufficient evidence to reject the null hypothesis.

## 3. Results

### 3.1. Facial Measurements

The mean upper facial height is 62.57 (SD = 6.678883). The value of the K-S test statistic (D) is 0.1409. The *p*-value is 0.77154. The data do not differ significantly from those which are normally distributed.

The mean bizygomatic width is 128.765 (SD = 5.451053). The value of the K-S test statistic (D) is 12,794. The *p*-value is 0.85841. The data do not differ significantly from those which are normally distributed.

The mean palatal length is 45.385 (SD = 4.552648). The value of the K-S test statistic (D) is 11639. The *p*-value is 0.9207. The data do not differ significantly from those which are normally distributed.

### 3.2. Volumes of the Left and Right Maxillary Sinuses

The differences between the left and right MS volumes reported in Table 2 are not statistically significant (*p*-value = 0.7368).

### 3.3. Linear Measurement along the Sagittal Axis of the Left and Right Maxillary Sinuses

The differences between the sagittal sizes of the left and right MSs reported in Table 3 are considered not statistically significant (*p*-value = 0.5256).

### 3.4. Linear Measurement along the Transverse Axis of the Left and Right Maxillary Sinuses

The differences between the transversal sizes of the left and right MSs reported in Table 4 are considered not statistically significant (*p*-value = 0.4897).

### 3.5. Linear Measurement along the Vertical Axis of the Left and Right Maxillary Sinuses

The differences between the transversal sizes of the left and right MSs reported in Table 5 are considered not statistically significant (*p*-value = 0.6950).

## 4. Discussion

Recently, CBCT has been more and more useful for descriptive and quantitative analysis of postnatal growth and development of the paranasal sinus [25].

Given the limited size of the sample, for which the central limit theorem does not hold, the Jarque–Bera and Shapiro–Wilk tests were used to assess the sample distribution. However, both tests yielded negative results. This indicates that the distribution of observations in the sample significantly deviates from the normal or Gaussian distribution. In other words, the nature of the data distribution does not align with the expectations of statistical normality.

This finding is noteworthy as the Jarque–Bera and Shapiro–Wilk tests are commonly employed to check for data normality. In this paper, the negative outcomes of both tests suggests that the data distribution in the sample does not follow the typical bell-shaped curve of a normal distribution. Therefore, the non-parametric statistical Wilcoxon test was calculated considering the constraints arising from the small sample size and the non-normality of the distribution.

This research compared the right and the left MSs in the 20 fossil crania discovered in the necropolis of Opi, in the middle of Italy, and did not find asymmetry between the left MS and right MS, contrary to what has been reported in the literature, where the difference between left and right MSs has usually been detected in modern individuals in terms of length and width; some authors found a suitable MS bigger than the left MS and vice versa, and other ones did not find this asymmetry in humans [26,27].

In 2010, the same population from Opi was previously examined in a paper focused on maxillary and mandibular base size. A significant difference was found by Festa et al. for the maxillary length, which was more important in the fossil cranium. The mandible was shorter in modern individuals but without significant differences compared to ancient skulls. These measurements were computed on lateral cephalograms [28].

As found in 2011, in a paper focused on the skeletal robusticity of the Samnites from Alfedena (Aq), men showed higher lateralisation, likely due to weapon training and a preferential use of the dominant arm. Alfedena men were three times more lateralised than modern Euroamericans, with values between professional tennis players and Palaeolithic hunters [29].

The maxillary sinus shape is significantly influenced by the changes in craniofacial morphology seen during the development of Hominidae.

The growth pattern of the MSs in the Japanese macaque (Macaca fuscata) has a structural role in the skull architecture, and a tight relationship was also demonstrated between MS volume and external cranial dimensions by Koppe et al.

As found by Buck et al., the MS volume in our species and H. sapiens was significantly smaller. It resulted in hypopneumatisation when compared to its closest congeners and other taxa [30].

Sinuses have a thermoregulatory role, and sinus volume decreases in cold temperatures.

The MSs in Eskimo populations were studied, and the analysis revealed strong correlations with the climate; in fact, MS volume in Eskimo populations decreases in colder areas [31].

Few similar studies have been conducted in the literature. Evteev et al. (2019) found a strong association between the form of the nasal cavity and climate across 15 populations of East Asian origin inhabiting the Far East, Siberia, Alaska, and Greenland [32].

Butaric et al. (2015) affirmed that nasal cavity size and shape are vital in air conditioning processes using CBCT in modern human crania from nine different geographic areas [33].

CBCT could be a promising technique for the sex and age determination of ancient skulls by analysing paranasal volume and size due to excellent resolution [34].

## 5. Conclusions

In none of the four cases did H1 occur. Therefore, there is no significant difference between the left and right maxillary sinuses regarding volume and individual measurements along the space axes.

Maxillary sinus measurements can aid in forensic anthropology to evaluate changes in the maxillary sinus area with human evolution over time.

Despite the limitations of the present research, the findings highlighted the anatomic variability in the MS.

Further studies with larger CBCT images recruiting all age and ethnic groups will be recommended for more information on the population initially from Opi.

## Figures and Tables

**Table 1:** Facial measurements.

	Upper Facial Height (mm)	Bizygomatic Width (mm)	Palatal Length (mm)
**OPI T14**	63.2	137.4	46.9
**OPI T22**	54.9	125.2	43.7
**OPI T31**	56.4	128.6	44.7
**OPI T34**	59.2	129.2	40.7
**OPI T37**	57.3	139.9	45.6
**OPI T41**	63.8	122.8	43.1
**OPI T47**	63.6	129.5	43.1
**OPI T57**	78.6	128.8	42.6
**OPI T59**	66.7	129.3	46.2
**OPI T60**	56.7	132.8	44.6
**OPI T63**	71.8	127.8	40.3
**OPI T64**	66.9	120.0	38.8
**OPI T65**	54.9	126.6	40.2
**OPI T76**	53.8	124.5	46.8
**OPI T86**	64.2	116.7	48.7
**OPI T87**	70.0	130.0	48.5
**OPI T90**	69.4	134.2	42.0
**OPI T91**	62.6	132.9	53.4
**OPI T95**	62.3	131.1	55.8
**OPI T104**	55.1	128.0	52.0

**Table 2:** Volumes of the left and right maxillary sinuses.

	Left MS Volume (mm^3^)	Right MS Volume (mm^3^)
**OPI T14**	33,175	28,840
**OPI T22**	21,727	22,039
**OPI T31**	21,344	21,486
**OPI T34**	19,269	18,687
**OPI T37**	24,451	25,155
**OPI T41**	23,594	20,474
**OPI T47**	22,845	22,016
**OPI T57**	21,299	22,071
**OPI T59**	18,615	17,529
**OPI T60**	14,071	15,288
**OPI T63**	21,125	12,491
**OPI T64**	19,083	21,482
**OPI T65**	14,485	13,944
**OPI T76**	25,852	26,975
**OPI T86**	20,577	19,302
**OPI T87**	21,603	26,149
**OPI T90**	15,024	16,873
**OPI T91**	23,513	20,745
**OPI T95**	18,726	16,873
**OPI T104**	21,727	26,264

**Table 3:** Linear measurements along the sagittal axis of the left and right maxillary sinuses.

	Left (mm)	Right (mm)
**OPI 14**	1514	1357
**OPI 22**	1078	1196
**OPI 31**	895	1067
**OPI 34**	1075	1097
**OPI 37**	1129	1274
**OPI 41**	1280	1102
**OPI 47**	1184	1147
**OPI 57**	1152	1135
**OPI 59**	1122	1148
**OPI 60**	932	947
**OPI 63**	1097	741
**OPI 64**	1000	1093
**OPI 65**	768	882
**OPI 76**	1193	1276
**OPI 86**	1124	999
**OPI 87**	910	1120
**OPI 90**	1015	1033
**OPI 91**	1216	1171
**OPI 95**	1097	1033
**OPI 104**	1078	1294

**Table 4:** Linear measurements along the transverse axis of the left and right maxillary sinuses.

	Left (mm)	Right (mm)
**OPI 14**	1020	1057
**OPI 22**	760	668
**OPI 31**	673	780
**OPI 34**	730	722
**OPI 37**	815	834
**OPI 41**	842	737
**OPI 47**	823	785
**OPI 57**	825	891
**OPI 59**	600	562
**OPI 60**	449	514
**OPI 63**	656	419
**OPI 64**	757	745
**OPI 65**	572	507
**OPI 76**	911	857
**OPI 86**	840	792
**OPI 87**	737	898
**OPI 90**	645	652
**OPI 91**	799	703
**OPI 95**	721	652
**OPI 104**	760	885

**Table 5:** Linear measurements along the vertical axis of the left and right maxillary sinuses.

	Left (mm)	Right (mm)
**OPI 14**	1035	873
**OPI 22**	890	830
**OPI 31**	867	764
**OPI 34**	709	737
**OPI 37**	898	852
**OPI 41**	941	841
**OPI 47**	760	795
**OPI 57**	735	811
**OPI 59**	793	721
**OPI 60**	600	712
**OPI 63**	904	621
**OPI 64**	742	860
**OPI 65**	637	594
**OPI 76**	836	924
**OPI 86**	687	665
**OPI 87**	964	996
**OPI 90**	576	671
**OPI 91**	799	767
**OPI 95**	652	671
**OPI 104**	890	915

## Data Availability

The data presented in this study are available on request from the corresponding author due to legal reasons.
